# Evidence That VirS Is a Receptor for the Signaling Peptide of the Clostridium perfringens Agr-like Quorum Sensing System

**DOI:** 10.1128/mBio.02219-20

**Published:** 2020-09-15

**Authors:** Jihong Li, Bruce A. McClane

**Affiliations:** a Department of Microbiology and Molecular Genetics, University of Pittsburgh School of Medicine, Pittsburgh, Pennsylvania, USA; University of Oklahoma Health Sciences Center

**Keywords:** *Clostridium perfringens*, beta toxin, Agr-like quorum sensing, VirS/R two-component regulatory system, signal peptide receptor, toxin production regulation

## Abstract

Since both the Agr (accessory gene regulator)-like quorum sensing (QS) system and VirS/VirR (VirS/R) two-component regulatory system of Clostridium perfringens positively regulate production of several toxins, including C. perfringens beta toxin (CPB), it has been hypothesized the VirS membrane sensor protein is an Agr-like QS signaling peptide (SP) receptor. To begin evaluating whether VirS is an SP receptor, this study sequenced the *virS* gene in C. perfringens strains CN3685 and CN1795 because it was reported that *agrB* mutants of both strains increase CPB production in response to the pentapeptide 5R, likely the natural SP, but only the CN3685 *agrB* mutant responds to 8R, which is 5R plus a 3-amino-acid tail. This sequencing identified differences between the predicted VirS extracellular loop 2 (ECL2) of CN3685 versus that of CN1795. To explore if those ECL2 differences explain strain-related variations in SP sensitivity and support VirS as an SP receptor, *virS agrB* double-null mutants of each strain were complemented to swap which VirS protein they produce. CPB Western blotting showed that this complementation changed the natural responsiveness of each strain to 8R. A pulldown experiment using biotin-5R demonstrated that VirS can bind SP. To further support VirS:SP binding and to identify a VirS binding site for SP, a 14-mer peptide corresponding to VirS ECL2 was synthesized. This ECL2 peptide inhibited 5R signaling to *agrB* mutant and wild-type strains. This inhibition was specific, since a single N to D substitution in the ECL2 peptide abrogated these effects. Collectively, these results support VirS as an important SP receptor and may assist development of therapeutics.

## Abstract (precis)

C. perfringens beta toxin (CPB) is essential for the virulence of type C strains, a common cause of fatal necrotizing enteritis and enterotoxemia in humans and domestic animals. Production of CPB, as well as several other C. perfringens toxins, is positively regulated by both the Agr-like QS system and the VirS/R two-component regulatory system. This study presents evidence that the VirS membrane sensor protein is a receptor for the AgrD-derived SP and that the second extracellular loop of VirS is important for SP binding. Understanding interactions between SP and VirS improves knowledge of C. perfringens pathogenicity and may provide insights for designing novel strategies to reduce C. perfringens toxin production during infections.

## INTRODUCTION

The Gram-positive, anaerobic bacterium Clostridium perfringens is a major cause of histotoxic and intestinal infections in humans and other animals (1–3). C. perfringens infections such as gas gangrene, enteritis, and enterotoxemia are mediated in large part by the ability of this bacterium to produce more than 20 different toxins (4, 5). However, individual strains produce only subsets of this toxin repertoire, allowing for a classification system based on carriage of genes encoding major toxins, i.e., C. perfringens alpha toxin (CPA), C. perfringens beta toxin (CPB), C. perfringens enterotoxin (CPE), and epsilon (ETX), iota (ITX), and NetB toxins. This scheme assigns C. perfringens isolates to one of seven types (A to G) (6). Of importance for the current study, type B isolates must carry genes encoding CPA, CPB, and ETX toxins, while type C isolates must carry only genes encoding CPA and CPB toxins. Each C. perfringens type is associated with particular diseases, e.g., type B and C strains cause necrotizing enteritis and enterotoxemia (1–3, 5).

Toxin production by pathogenic bacteria is often regulated by quorum sensing (QS) systems and two-component regulatory systems (TCRS), which can work in tandem (7, 8). Consistent with that general theme, C. perfringens toxin production is frequently regulated by both TCRS and QS systems (9). The two most common regulatory systems for controlling toxin production in C. perfringens are the VirS/VirR (VirS/R) TCRS and the accessory gene-like regulator (Agr) QS system (9–11).

The VirS/R TCRS consists of the membrane sensor histidine kinase VirS and the response regulator VirR (12). This system, encoded by the *virR virS* (*virR/S*) operon, becomes activated by a signal that induces autophosphorylation of VirS, which is followed by phosphotransfer to VirR (13). Phosphorylated VirR can directly regulate expression of some C. perfringens toxin genes, such as the *netB* gene encoding NetB or the *pfoA* gene encoding the perfringolysin O (PFO), by binding to VirR boxes located upstream of the promoters for those genes (14–17). Alternatively, phosphorylated VirR indirectly regulates other C. perfringens toxin genes (such as *cpa*) by inducing expression of a small regulatory RNA named VR-RNA (18). The VirS/R TCRS can be important for C. perfringens virulence. For example, this TCRS was shown to be essential for type C strain CN3685 to cause intestinal pathology in rabbit small intestinal loops or enterotoxemic lethality in mice (19). Consistent with this pathogenic role, the VirS/R TCRS was found to regulate CPB production *in vivo* (19), which is important since CPB plays a critical role when type C strains cause necrotic enteritis or enterotoxemia (20).

The Agr QS system was first discovered in Staphylococcus aureus, where it controls expression of genes encoding many toxins and degradative enzymes in a quorum-sensing manner (8, 21). In S. aureus, an autoinducing signaling peptide (AIP) is encoded by the *agrD* gene and modified and secreted by the AgrB membrane protein. In the same S. aureus operon encoding AgrB/AgrD (AgrB/D) are genes encoding the AgrC/AgrA (AgrC/A) TCRS. In this system, AgrC is a sensor receptor protein for the AIP and acts by phosphorylating the transcriptional regulator AgrA to induce expression of a gene, located upstream of the *agr* operon, that encodes a small regulatory RNA named RNAIII. RNAIII then regulates toxin production (8, 21, 22).

Interestingly, while C. perfringens has an operon encoding AgrB and AgrD, genes encoding AgrC/A or RNAIII are not present in the C. perfringens genome (23, 24). Nonetheless, the C. perfringens Agr-like QS system regulates the production of several toxins, such as PFO, CPA, CPB, and NetB (23–26). Consequently, the Agr-like QS system is an important virulence regulator. For example, this QS system was shown to be necessary for the pathogenicity of both type C and NetB-positive type G strains (25, 26).

Our previous study (27) showed that C. perfringens type C strain CN3685 and type B strain CN1795 *agrB* mutants, which carry an intron insertion in their *agrB* gene and do not express the operon encoding AgrB and AgrD, differ in their sensitivity to different synthetic C. perfringens signal peptide (SP)-based peptides. For both strains, their *agrB* mutants respond to 5R, which likely corresponds to the natural 5-mer SP of C. perfringens (27), while only the CN3685 *agrB* mutant can respond to 8R, which is SP plus a 3-amino-acid tail (27).

Since production of several C. perfringens toxins, including CPB, is positively regulated by both the Agr-like QS system and the VirR/S TCRS, it has been suggested that the VirS membrane protein is an SP receptor (9, 23). However, this correlation has not yet been supported by any direct experimental evidence indicating that the SP can bind to and signal VirS. In this study, we provide several lines of evidence that the SP interacts with and signals VirS and that this process involves SP binding to the main extracellular loop of VirS.

## RESULTS

### Quantitation of *agrD*, *virS*, and *cpb* expression by real-time qRT-PCR.

Previous studies (19, 25, 28, 29) showed that (i) CPB toxin production is regulated by both the Agr-like QS system and the VirS/R TCRS and (ii) CPB is generally produced in the early stationary phase when C. perfringens populations had achieved high cell density. Therefore, this study first examined the linkage between *cpb* transcript levels and *agrD* or *virS* expression levels using reverse transcription-quantitative PCR (qRT-PCR).

**FIG 1:**
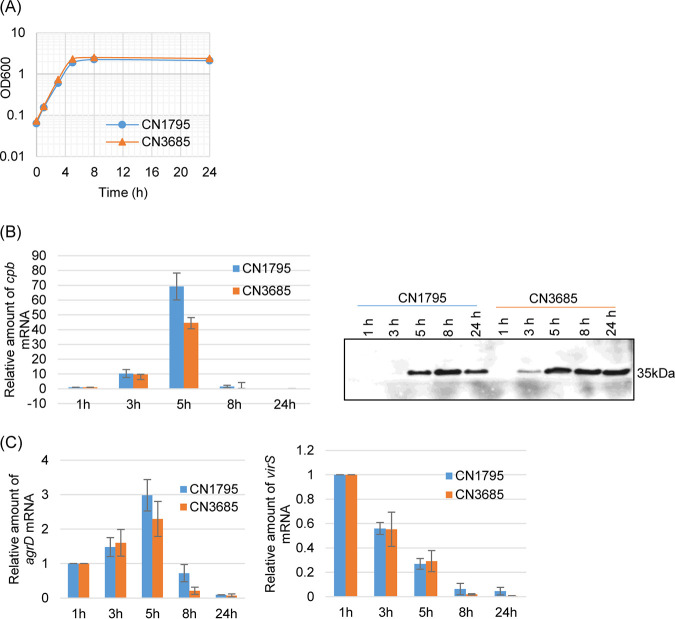
Growth curves and timing of Clostridium perfringens beta toxin (CPB) production versus *virS*, *agrD*, and *cpb* expression in wild-type CN1795 and CN3685 cultures. (A) Growth curves for wild-type CN1795 and CN3685 strains cultured at 37°C for up to 24 h in TY medium. Aliquots of each culture were measured for their optical density at 600 nm (OD_600_) at 1, 3, 5, 8, 10, and 24 h. This experiment was repeated three times, and a representative result is shown. (B) Reverse transcription-quantitative PCR (qRT-PCR) analyses of *cpb* expression levels (left) and CPB Western blot analysis (right) for CN1795 and CN3685. Transcript levels were determined using 10 ng of RNA isolated from TY cultures of these strains grown for 1, 3, 5, 8, 10, and 24 h at 37°C. Average threshold cycle (*C_T_*) values were normalized to that of the 16S rRNA housekeeping gene, and fold differences were calculated using the comparative *C_T_* method (2^−ΔΔ^*^CT^*). Values of each bar indicate the fold change versus the 1-h culture. Western blot analysis for CPB production in supernatants of the two wild-type strains cultured in TY culture for 1, 3, 5, 8, 10, and 24 h. (C) qRT-PCR analyses of *agrD* and *virS* expression levels using the same cDNA preparation as that used for measuring *cpb* transcripts. Quantitative RT-PCR analyses shown in panels B and C were repeated three times, and mean values are shown. Error bars indicate standard deviations. A representative CPB Western blot based upon 3 repetitions is shown.

For this analysis, growth curves were first determined (Fig. 1A) for a type B strain (CN1795) and a type C strain (CN3685) cultured in TY medium, and production of CPB in those same cultures was also evaluated by Western blotting at various time points (Fig. 1B). The results showed that both strains grew similarly and that their CPB production began 3 h after inoculation (during the logarithmic growth phase). The production of CPB then increased further until 8 h (during the stationary phase). Compared to 1 h after inoculation, *cpb* gene transcript levels had increased by 3 h postinoculation and then reached maximum levels at 5 h postinoculation (during the early stationary phase). Levels of the *cpb* gene transcript were barely detected in 8-h (stationary-phase) or overnight (16-h, death phase) cultures (Fig. 1B).

Using the same cDNA samples, *agrD* and *virS* transcript levels were also determined by qRT-PCR. The results showed that the *agrD* transcript level pattern was very similar to the *cpb* expression pattern (Fig. 1C, left). However, *virS* gene transcript levels were maximally expressed at 1 h postinoculation and then decreased thereafter (Fig. 1C, right). The patterns of *agrD* or *virS* expression were very similar in both C. perfringens strains examined.

### Sequence differences between the *virS* gene in CN1795 versus that in CN3685.

**FIG 2:**
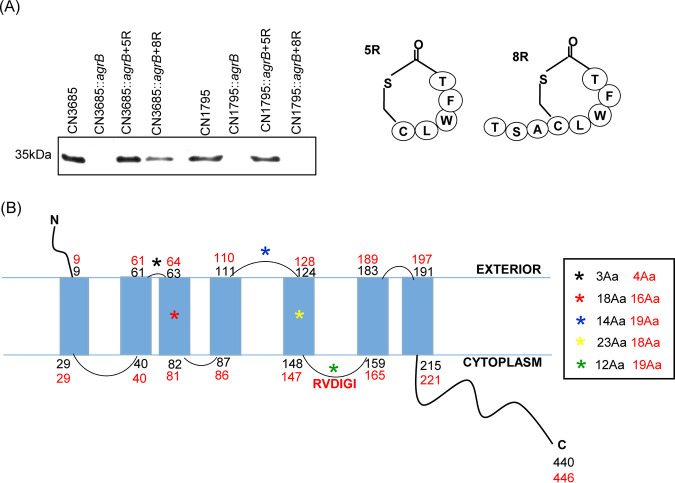
Modeling of the VirS proteins produced by CN1795 versus CN3685. (A) Comparison of signaling sensitivity of CN1795 and CN3658 to SP-based peptides 5R and 8R. CPB production (left) by wild-type or *agrB* null mutant (*agrB*KO) strains of CN3685 and CN1795 in the presence of 100 μM 5R and 8R (structures shown in the right panel). The samples for CPB Western blotting were prepared from the supernatants of overnight TY cultures (about 16 h) at 37°C. (B) The predicted topology of VirS protein produced by two wild-type strains. The predicted CN1795 VirS structure is shown in black numbers (total of 440 amino acids). The predicted CN3685 VirS structure is shown in red numbers (total of 446 amino acids). Asterisks (*) with different colors show the differences in this VirS region between these two strains. Amino acid (Aa) differences (indicated by asterisks) between the two strains are shown on the right side, with amino acid sequence numbers for CN1795 shown in black and those of CN3685 shown in red.

Our lab previously prepared *agrB* mutants of type B strain CN1795 and type C strain CN3685 (25, 30). These mutants have an intron insertion in their *agrB* gene that inactivates expression of both the *agrB* and a*grD* genes, which are located in the same operon (24, 25, 30). We also showed previously (27) that those CN1795 and CN3685 *agrB* mutants respond to SP-based synthetic peptides (Fig. 2A) named 5R (a 5-amino-acid thiolactone ring that likely corresponds to the natural C. perfringens SP [27]), but only the CN3685 *agrB* mutant responds to 8R, which is 5R plus a 3-amino-acid tail. The current study first confirmed those previous observations using newly synthesized 5R and 8R peptides. The results, shown in Fig. 2A, indicated that the CN1795 *agrB* null mutant only responded to the 5R peptide. In contrast, the CN3685 *agrB* null mutant responded to both the 5R and 8R peptides, although there was stronger signaling (more CPB production) with the 5R versus the 8R peptide, consistent with our previous study (27).

Since it has been hypothesized (9) that the VirS membrane sensor protein of the VirS/R TCRS is a receptor for the AgrD signaling peptide, and the two *agrB* null mutant strains respond differently to the 5R or 8R peptides, we compared the VirS proteins made by these two strains using *virS* sequencing. Differences detected between the deduced VirS sequences of these two strains are shown in Table 1. Those differences included 3 single amino acid differences at residues 81, 360, and 382 (relative to the CN1795 VirS sequence). However, the most striking difference noted between the *virS* open reading frames (ORFs) of these two strains is that the *virS* ORF in CN3685 encodes a 6-amino-acid insertion at the 152nd amino acid, producing a VirS protein of 446 amino acids compared to the 440-amino-acid VirS protein made by CN1795.

**TABLE 1:** Sequence differences between the VirS proteins of CN1795 and CN3685

Strain	Difference(s) at VirS amino acid position^*a*^:			
	81	152	360	382
CN1795VirS	R		S	I
CN3685VirS	K	RVDIGI	N	V

a Amino acid numbers shown refer to the amino acid position in the CN1795 VirS protein.

The transmembrane prediction algorithm (TMHMM) predicts that the VirS protein has 4 extracellular regions that might interact with SP if VirS is an SP receptor (Fig. 2B). Furthermore, the TMHMM program predicts that the sequence differences detected between CN3685 versus CN1795 impact VirS structure, as shown in Fig. 2B. Of particular interest for this study, these sequence differences are predicted to result in an extracellular loop 2 (ECL2) of 14 amino acids for the CN1795 VirS protein versus an ECL2 of 19 amino acids for the CN3685 VirS protein (Fig. 2B, blue asterisk).

### Preparation of *virS agrB* double-null mutant strains of CN1795 or CN3685 and swapping of the *virR/S* operon expressed by those double-null mutant strains.

Given the differences in 8R signaling sensitivity observed between CN1795 and CN3685 (Fig. 2A; see also reference 27) and the predicted structural differences in their VirS proteins (Fig. 2B), we inactivated the *virS* gene in our existing *agrB* mutants and then complemented those double mutants to swap which VirS protein they expressed in order to test if this swap affects 8R sensitivity, as might be anticipated if VirS is an SP receptor.

**FIG 3:**
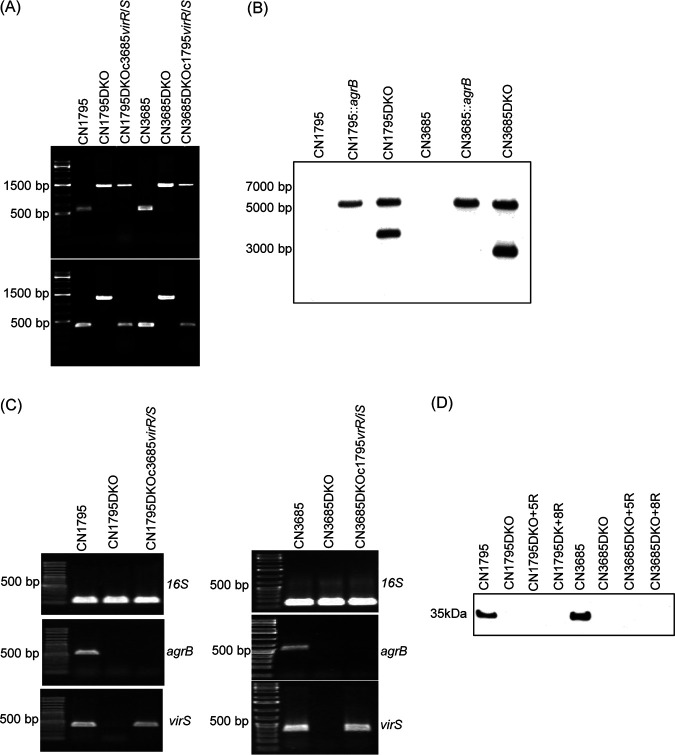
Preparation and characterization of CN1795 or CN3685 *agrB virS* double-null mutants and complementation of those double mutants to express a swapped VirS. (A) PCR confirmation of *agrB virS* double-null mutant construction by targeted intron-based mutagenesis and genetic complementation of those mutants with a swapped *virR/S* operon. Using DNA isolated from wild-type strains, a PCR assay amplified ∼500-bp products using internal *agrB* primers (upper) or internal *virS* primers (lower). After targeted insertion of a 900-bp intron, the same PCR assays amplified an ∼1.5-kb product using DNA isolated from the double-null mutant (DKO) strains. After complementation of the double mutants to carry the swapped *virR/S* operon (creating CN1795DKOc3685*virR/S* and CN3685DKOc1795*virR/S*), *agrB* PCR products remained the large size indicative of an intron insertion, but the *virS* PCR products were the smaller size present in wild-type strains, confirming genetic *virS* complementation. A 1-kb molecular ladder (Fisher Scientific) was used, with the size in bp shown at left. (B) Southern blot hybridization of an intron-specific probe with DNA from wild-type strains, the *agrB* single mutants, or *agrB virS* double-null mutants. DNA from each strain was digested with EcoRI and electrophoresed on a 1% agarose gel prior to blotting and hybridization with an intron-specific probe. Size of DNA fragments, in kb is shown at left. (C) RT-PCR analyses for expression of 16S RNA (top), the *agrB* gene (middle), or the *virS* gene (bottom) by wild-type CN1795 (left) or CN3685 (right), their *agrB virS* double-null mutants (DKO), or complementing strains of those double mutants with a swapped *virR/S* operon. Those samples did not amplify a product when subjected to PCR without reverse transcription, indicating that the RNA preparations from all strains were free from DNA contamination (data not shown). (D) Western blot analyses of CPB production by wild-type strains, *agrB virS* double-null mutants, or those mutants cultured in the presence of 100 μM 5R or 8R peptides. Size of proteins in kDa is shown at left. All experiments were repeated three times, and representative results of three repetitions are shown.

For this purpose, a Clostridium-modified TargeTron insertional mutagenesis method (31) was employed to introduce a targeted intron into the *virS* gene of CN1795*agrB*KO and CN3685*agrB*KO to create *virS agrB* double-null mutants that do not express either the *virS* gene or the operon encoding *agrB/D*. The identity of those putative double mutants, named CN1795DKO and CN3685DKO, was first demonstrated by PCR using primers specific for internal *agrB* open reading frame (ORF) or *virS* ORF sequences. Compared to the PCR products amplified from the *agrB* or *virS* genes in wild-type strains, PCR of DNA from the double mutants amplified larger bands from both genes due to insertion of an intron into the wild-type *agrB* and *virS* genes (Fig. 3A).

To create complementing strains producing a swapped VirS protein (e.g., CN1795 producing the VirS protein of CN3685), the *virR/S* operons of CN1795 or CN3685 were separately cloned into the pJIR750 shuttle plasmid. The plasmid carrying the CN1795 *virR/S* operon was then electroporated into CN3685DKO, and the plasmid carrying the CN3685 *virR/S* operon was electroporated into CN1795DKO, creating (respectively) two complementing strains named CN3685DKOc1795*virR/S* and CN1795DKOc3685*virR*/*S* that now expressed the *virR/S* operon of the other strain (Fig. 3A). PCR results confirmed that, in these two complementing strains, the presence of a wild-type *virS* gene was restored. Furthermore, an intron-specific Southern blot analysis (Fig. 3B) demonstrated that, while only one intron is present in the *agrB* single mutant strains, the double mutant strains now carry two intron insertions. RT-PCR results (Fig. 3C) demonstrated that, in the double-null mutant strains, neither the *agrB* nor the *virS* gene was expressed, while the swapped *virR/S* complementing strains exhibited restored *virS* gene expression. Western blot results (Fig. 3D) confirmed that CPB, which is regulated by both the Agr-like QS system and the VirS/R TCRS, was not expressed in either double-null mutant strain, even when the 5R signaling peptide was added.

**FIG 4:**
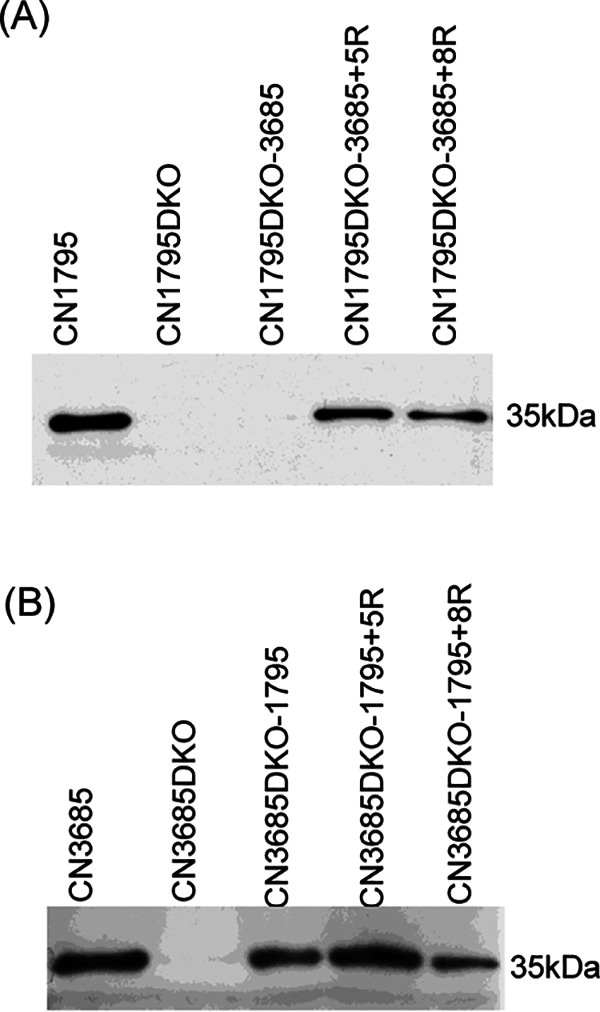
Western blot analyses of CPB production by wild-type, *agrB virS* double-null mutant, and swapped complementing strains, grown in the presence or absence of 100 μM 5R or 8R peptide. Cultures were grown for 5 h at 37°C in TY medium, and the OD_600_ values of each culture were adjusted to the same density. Supernatants were then collected, and equal volumes were subjected to CPB Western blotting. Size of proteins in kDa is shown at left. All experiments were repeated three times, and results representative of three repetitions are shown.

The main purpose of constructing these complementing strains producing a swapped VirS was to test their responsiveness to signaling by peptides 5R or 8R, as assessed by CPB Western blot analysis (Fig. 4). The CN1795 complementing strain expressing CN3685 VirS responded to both the 5R and 8R peptides, similarly to CN3685 *agrB* knockout (KO) but unlike CN1795 *agrB* KO. However, the CN3685 complementing strain expressing CN1795 VirS responded only to the 5R peptide, unlike CN3685 *agrB* KO but resembling CN1795 *agrB* KO. While this complementing strain also expressed trace amounts of CPB even without addition of a signaling peptide, likely attributable to VirR overproduction due to complementation with a multicopy plasmid (13), this CPB production did not increase further when supplied the 8R peptide. We also tested CPB production by this complementing strain when incubated with or without 8R for different time points (3 h, 5 h, 8 h, or overnight), but the presence of 8R did not increase CPB production compared to the absence of 8R at any time point (data not shown).

### Evidence that a biotin-labeled 5R peptide (B-5R) can bind to the His_6_-tagged VirS protein of CN3685.

Attempts to prepare a VirS antibody for use in a biotin-labeled 5R peptide (B-5R) pulldown experiment were unsuccessful. Therefore, we used a CN3685 strain producing His_6_-tagged VirS and a His_6_ antibody Western blot to determine, in a pulldown assay, whether VirS can physically bind with B-5R, as would be expected if VirS is an SP receptor in C. perfringens.

**FIG 5:**
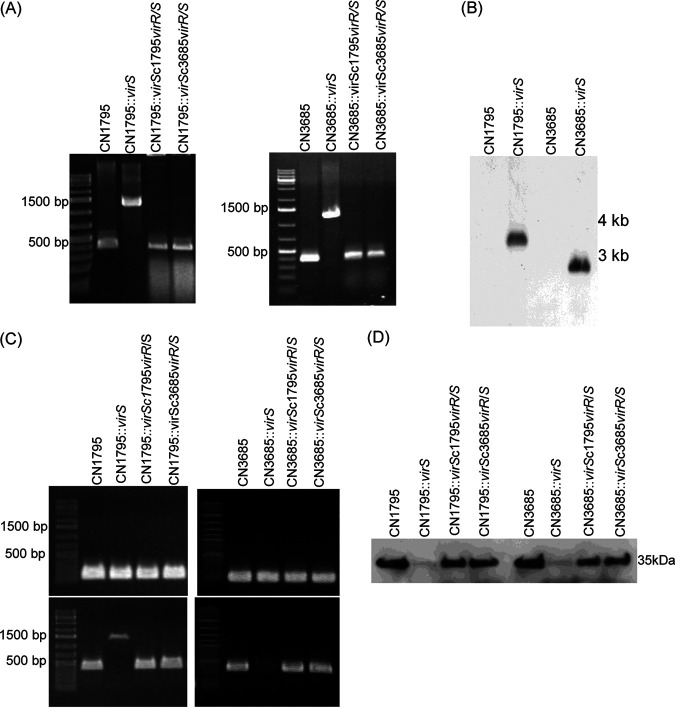
Preparation and characterization of CN1795 or CN3685 *virS* null mutants and complemented strains. (A) PCR confirmation of *virS* null mutants created by intron-based mutagenesis or complementing strains carrying a *virR/S* operon from CN1795 or CN3685 (indicated by c1795*virR/S* or c3685*virR/S*). Using DNA isolated from wild-type strains, an ∼500-bp product was PCR amplified using internal *virS* primers. However, after targeted insertion of a 900-bp intron into *virS*, the same PCR assay amplified an ∼1.5-kb product using DNA from the null mutant strains. Using DNA from the complemented strains, the same-size *virS* PCR products were amplified as when using DNA from wild-type strains. A 1-kb molecular ladder (Fisher Scientific) was also electrophoresed, and the size in bp is shown at left. (B) Southern blot hybridization of an intron-specific probe with DNA from wild type or the *virS* null mutants of CN1795 or CN3685. DNA from each strain was digested with EcoRI and then electrophoresed on a 1% agarose gel prior to blotting and hybridization with an intron-specific probe. Size of DNA fragments in kb is shown at right. (C) RT-PCR analyses of 16S RNA (top) and *virS* (bottom) transcription in wild-type CN1795 (left) or CN3685 (right), their *virS* null mutants, or complementing strains carrying the *virR/S* operon of either strain. To show that the RNA preparations from both strains were free from DNA contamination, the samples were also subjected to PCR without reverse transcription, but no products were amplified (data not shown). (D) Western blot analyses of CPB expression by wild type, *virS* null mutants, and complementing strains. Size of proteins in kDa is shown at left. All experiments were repeated three times, and results representative of three repetitions are shown.

For this purpose, the *virS* gene in CN3685 was inactivated by insertion of an intron using the *Clostridium*-modified TargeTron insertional mutagenesis method (31). Construction and characterization of this mutant, named CN3685::*virS*, is shown in Fig. 5. Specifically, PCR analyses (Fig. 5A) demonstrated the insertion of an ∼900-bp intron into the *virS* gene, since the PCR product amplified from this mutant strain DNA was 900 bp larger than the PCR product amplified from the wild-type CN3685 *virS* gene. An intron-specific Southern blot analysis (Fig. 5B) confirmed the presence of only one intron in this mutant.

Complementing strains named CN3685::*virSc*3685*virR*/*S* and CN3685::*virS*c1795*virR/S* were prepared that produce, respectively, the VirS of either CN3685 or CN1795. As expected, DNA from these complementing strains supported amplification of a PCR product of the same size as the wild-type *virS* gene (Fig. 5A). RT-PCR (Fig. 5C) analyses detected no *virS* gene expression by the mutant, while the two *virR/S* complementing strains showed *virS* gene expression. Western blot analyses (Fig. 5D) detected no CPB production by the CN3685 *virS* null mutant strain, while both complementing strains did produce CPB, confirming that their complemented VirS expression enabled functional signaling. At the same time, we similarly prepared a CN1795 null mutant (CN1795::*virS*) with an intron insertion in *virS* and a complementing strain producing the CN1795 VirS for studies (described later in Fig. 8).

For use in the pulldown experiment, we also transformed the CN3685 *virS* null mutant to produce VirS protein with a C-terminal His_6_ tag, creating CN3685::*virS*c3685*virR*/*S*his; the only difference between this complementing strain and CN3685::*virS*c3685*virR/S* is that a His_6_ tag was added to the VirS C terminus using a 5′-to-3′ PCR primer. For CN3685::*virS*c3685*virR/S*his, CPB Western blotting detected similar CPB production to that observed for CN3685::*virS*c3685*virR/S* (data not shown), confirming that the presence of the His_6_ tag did not interfere with VirS function.

**FIG 6:**
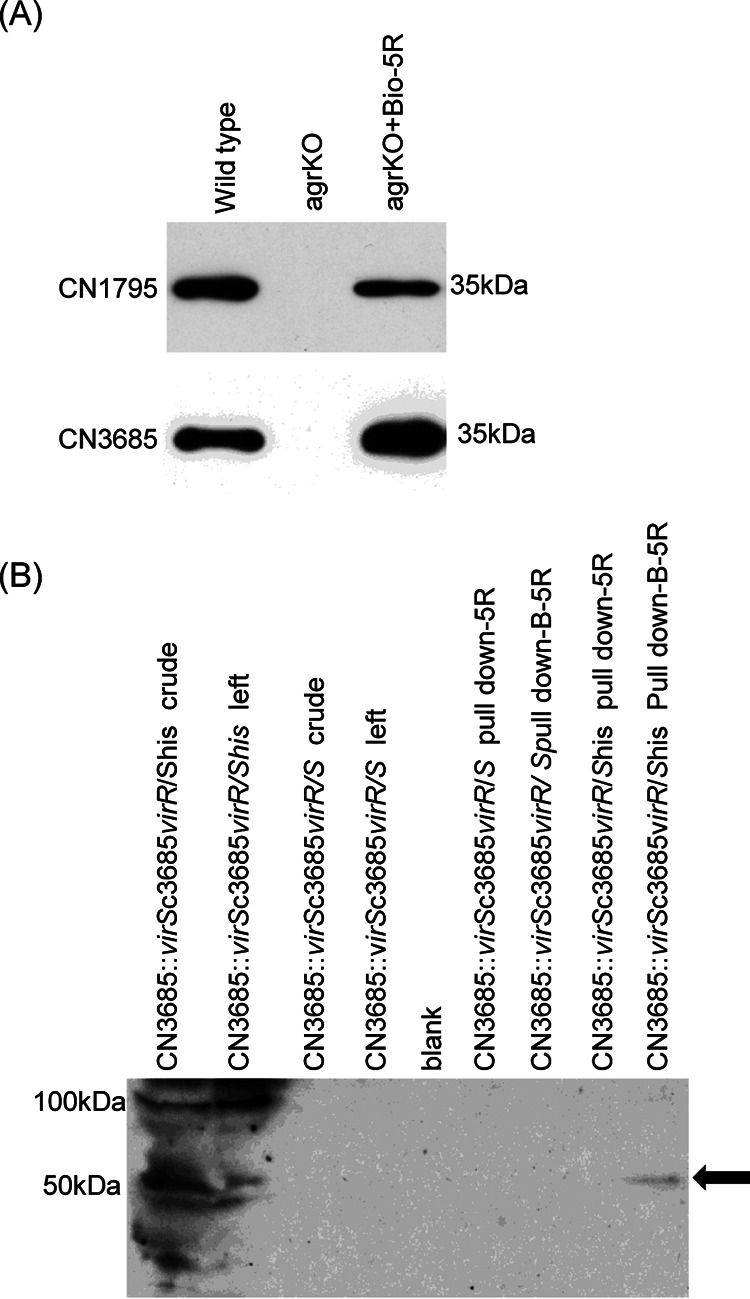
Streptavidin beads containing bound B-5R pull down His_6_-tagged VirS. (A) CPB production by wild-type or *agrB* mutant CN1795 (upper), wild-type or *agrB* mutant CN3685 (lower), or those null mutants incubated in the presence of 100 μM B-5R. Size of proteins in kDa is shown at left. (B) His_6_ tag Western blot of B-PER buffer extracts of CN3685::*virS*c3685*virR*/*Shis* (lane 1) or CN3685::*virS*c3685*virR/S* (lane 3). After those extracts were incubated with streptavidin beads preincubated with B-5R or 5R, unbound supernatant material is shown in lanes 2 and 4. Pulldowns of extracts from complementing strains using beads preincubated with 5R or B-5R are shown in lanes 5 to 9. Note that in these lanes, using CN3685::*virS*c3685*virR*/*S*his-containing extracts, but not CN3685::*virS*c3685*virR*/*S*-containing extracts, incubation of beads pretreated with B-5R, but not with 5R, resulted in pulldown of a protein that reacted with His_6_ tag antibody and was ∼50 kDa, the size of VirS. Size of protein markers in kDa is shown at left. All experiments were repeated three times, and results shown are representative of three repetitions.

Before using a biotin-labeled 5R peptide (B-5R) in a pulldown experiment to test for its interactions with VirS, it was important to evaluate whether this biotin-modified peptide retains biologic activity, i.e., signaling function. Western blot analysis showed that B-5R induced CPB production by both the CN1795 and CN3685 *agrB* null mutant strains (Fig. 6A).

With positive results from those control experiments, we then performed a pulldown experiment to evaluate if VirS can physically bind to SP. This involved preincubating streptavidin-coated beads with B-5R or 5R before mixing those beads with B-PER buffer cell extracts from 4-h TY cultures of CN3685::*virS*c3685*virR/S*his cells (Fig. 6B, lane 1). When those extracts were Western blotted with His_6_-specific antibody prior to incubation with the B-5R- or 5R-treated beads, a major immunoreactive band was detected that matched the ∼50-kDa size of VirS, although higher molecular mass species were also present, including an ∼100-kDa species that is likely a VirS dimer. After incubation of those extracts with the B-5R-coated streptavidin beads, much of the His_6_ antibody-immunoreactive material, particularly the apparent dimer, remained unbound in the supernatant (lane 2).

As a specificity control, Western blotting with His_6_ tag antibody detected (lane 3) no immunoreactive material in the crude extract of a control complementing strain (CN3685::*virS*c3685*virS*/*R*) that does not produce His_6_-tagged VirS, confirming that the immunoreactive material present in CN3685::*virS*c3685*virR*/*S*his extracts was attributable to His_6_-tagged VirS. In addition, no immunoreactive material was detected by His_6_ antibody using extracts of CN3685::*virS*c3685*virR/S* after pulldown by beads preincubated with either 5R or B-5R (lanes 6 and 7). However, an ∼50-kDa immunoreactive species was pulled down from extracts of CN3685::*virS*c3685*virR*/*S*his using beads preincubated with B-5R, which contains biotin and binds well to streptavidin-coated beads, but not from extracts of those cells preincubated with 5R, which lacks biotin and thus does not bind well to streptavidin-coated beads. This Western blot result analysis supports the ability of C. perfringens VirS to physically bind SP.

### A synthetic peptide corresponding to the predicted VirS second extracellular loop inhibits SP-induced CPB production.

The ability of B-5R-coated beads to pulldown VirS supports the binding of SP to VirS as a receptor. To confirm binding interactions between 5R and VirS by an independent approach and to discern a region of VirS involved in SP binding, we used the VirS structural model (Fig. 2B) to identify predicted extracellular regions of the VirS protein that could interact with SP. Since the predicted VirS ECL2 of CN1795 and CN3685 have significant size differences and those strains respond differently to signaling by 8R (Fig. 2), we hypothesized that this VirS loop is involved in SP binding. To test this hypothesis, we synthesized a 14-amino-acid peptide named KIGK (Table 2) that corresponds to the sequence of the predicted ECL2 of CN1795 VirS, as well as a random sequence control peptide with a molecular weight similar to that of KIGK.

**TABLE 2:** VirS ECL2 synthetic peptide sequences

Peptide	Sequence^*a*^	Daltons
KIGK	NH2-KINSLV**N**VSELLGK-CO2H	1,513.80
Control	NH2-AYSSGAPPMPPFFF-CO2H	1,515
KIGK_D	NH2-KINSLV**D**VSELLGK-CO2H	1,514.78

a Bolded residues show the single different amino acid residue present in KIGK versus KIGK_D.

**FIG 7:**
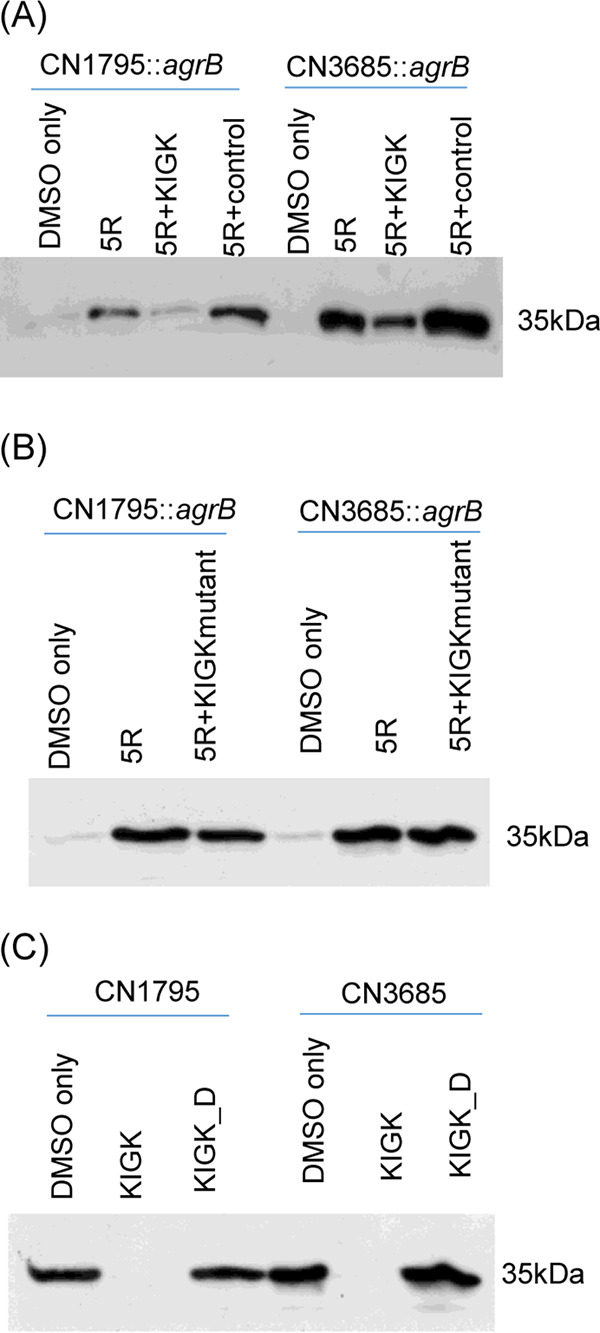
The KIGK peptide corresponding to the major predicted VirS ECL2 inhibits the ability of the 5R SP to induce CPB production. (A) Western blot showing effects of KIGK preincubation with 5R on CPB production by *agrB* null mutant strains of CN1795 and CN3685. Cultures of these strains were grown for 5 h at 37°C in TY medium with DMSO, DMSO plus 5R (100 μM), or DMSO plus 5R (100 μM) that had been preincubated with KIGK (500 M). (B) Western blot showing effects of preincubation of 5R with the KIGK_D peptide, which corresponds to the mutated VirS 2nd ECL, on CPB production by *agrB* null mutant strains of CN1795 and CN3685. Cultures of those strains were grown for 5 h at 37°C in TY medium with DMSO, DMSO plus 5R (100 μM), or DMSO plus 5R (100 μM) that had been preincubated with the KIGK mutant peptide (KIGK_D; 500 μM). (C) Western blot showing effects of KIGK on CPB production by wild-type CN1795 and CN3685 cultures. Cultures of those strains were grown in TY medium with DMSO, DMSO plus KIGK (1 mM), or DMSO plus KIGK_D (1 mM) for 5 h at 37°C. For all panels, CPB Western blots of culture supernatants are shown. Size of proteins in kDa is shown at left. All experiments were repeated three times, and results representative of three repetitions are shown.

To test if 5R can bind to KIGK, as might be anticipated if Agr-like QS signaling involves SP binding to the predicted ECL2 of VirS, the 5R and KIGK peptides were preincubated together before their addition to cultures of CN1795 or CN3685 *agrB* null mutant strains. CPB Western blot analysis (Fig. 7A) showed that neither *agrB* null mutant strain produces CPB in the absence of 5R, as expected from the results shown in Fig. 2. However, consistent with the hypothesis that 5R binds to the second ECL of VirS, preincubation of 5R with KIGK blocked Agr-like QS signaling for the *agrB* null mutants, i.e., CPB production was significantly reduced. In contrast, similar preincubation of 5R with the control peptide did not block AIP signaling to *agrB* null mutant strains, i.e., strong CPB production was detected. To further confirm the specificity of the binding interaction between 5R and KIGK, a peptide named KIGK_D was prepared where the N residue located in the middle of the predicted VirS second extracellular loop was switched to D (Table 2). Preincubation of 5R with this peptide had no effect on subsequent AIP signaling to *agrB* mutants, i.e., CPB production was not reduced (Fig. 7B).

To further test our hypothesis that SP binding involves the ECL2 of VirS, we applied KIGK (500 μM or 1 mM) to CN1795 and CN3685 to determine if this peptide can also block Agr-like QS signaling by those two wild-type strains. Results (Fig. 7C) showed that the presence of 1 mM KIGK peptide efficiently blocked CPB production by both wild-type strains, while the same dose of the KIGK_D peptide had no effect on CPB production. A 500 μM concentration of KIGK can partially block CPB production by these strains (data not shown).

### Cross-talk regulation of expression between the *virR/S* operon and the *agrB/D* operon.

**FIG 8:**
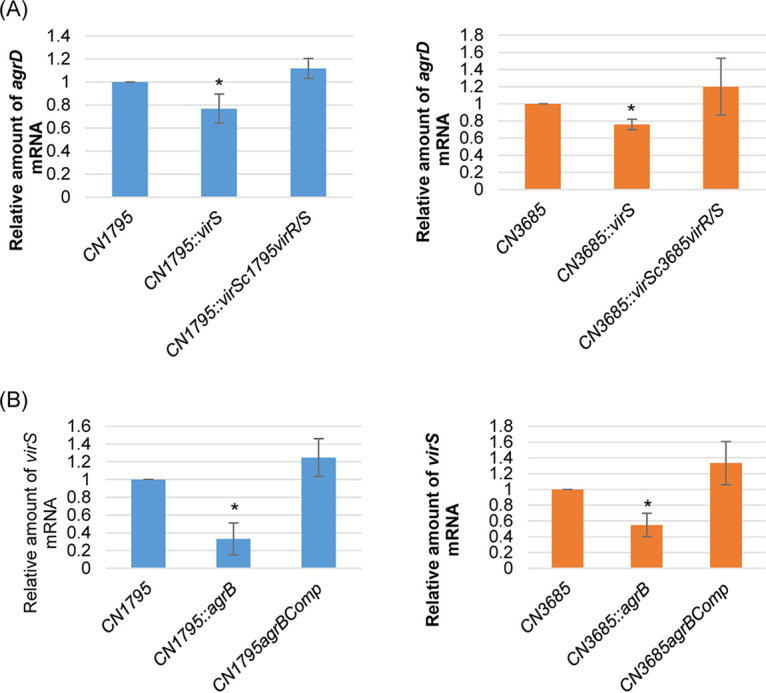
Quantitative RT-PCR analysis for *virS* and *agrD* gene expression in CN1795 (left) or CN3685 (right) wild-type, *agrB*, or *virS* null mutants, and complemented strains. Quantitative RT-PCR analyses of *agrD* (A) and *virS* (B) transcript levels were performed with 20 ng of the RNA isolated from 5-h (for *agrD* analyses) or 2-h (for *virS* analyses) TY medium cultures of the wild type, *virS* or *agrB* null mutants, and their complemented strains (comp). Average *C_T_* values were normalized to the value for the housekeeping 16S RNA gene, and the fold differences were calculated using the comparative *C_T_* (2^−ΔΔ^*^CT^*) method. The value of each bar indicates the calculated fold change relative to the value for the wild-type strains. Shown are the mean values from three independent experiments. *, *P* < 0.05 compared to the wild-type culture by ordinary one-way analysis of variance (ANOVA).

In S. aureus, sensing of the AIP causes the receptor AgrC to undergo histidine autophosphorylation and then phosphorylate AgrA, which initiates a positive feedback loop to make more AgrB and AgrD (21). Since the results shown in Fig. 3 to 7 provided substantial evidence supporting VirS as an SP receptor, we tested whether a positive feedback loop also functions in C. perfringens to help regulate expression of the Agr-like QS system and the VirS/R TCRS. This first involved using *virS* null mutant strains to study if the presence of VirS, an SP receptor, affects *agrD* expression. Based upon the kinetics for *agrD* expression determined in Fig. 1, expression levels of the *agrD* gene in 5-h TY medium cultures were compared between the two wild-type strains CN1795 and CN3685, as well as cultures of their *virS* null mutants and their *virR/S* operon-complemented strains. Results from these qRT-PCR analyses revealed significantly higher *agrD* transcript levels in the wild type and in its *virS/R*-complemented strain than in the *virS* null mutant strains (Fig. 8A).

To investigate whether SP production affects *virS* expression, the CN1795 and CN3685 *agrB* null mutant strains were used. Note that since *agrB* and *agrD* (and two upstream genes) are located within the same operon, the *agrD* gene is not expressed in null mutant strains with an insertion in their *agrB* gene (23, 24). Because *virS* expression levels peak early, i.e., at 1 to 2 h (Fig. 1), transcript levels of the *virS* gene were compared in 2-h TY medium cultures for both wild-type strains, their *agrB* null mutants, and those mutants complemented with the *agr* operon. Those qRT-PCR analyses detected significantly higher *virS* transcript levels in the wild type and its *agr* operon-complemented strains compared to those in the *agrB* null mutant strains (Fig. 8B).

## DISCUSSION

Producing toxins such as CPB is critical for C. perfringens pathogenicity (20, 32). Previous studies established that regulation of toxin production, including CPB production (19, 25), in C. perfringens often involves both the Agr-like QS system and the VirS/R TCRS. However, major gaps have remained in understanding of how those systems regulate C. perfringens toxin production, e.g., do these systems work cooperatively or independently? In comparison to the *agr* operon of the S. aureus Agr system, the C. perfringens *agr-*like operon encodes a different SP and does not encode a homolog of AgrC (11, 23, 24), which is the SP receptor in the S. aureus Agr QS system (11). Instead, since both the Agr-like QS system and VirS/R TCRS positively regulate production of several C. perfringens toxins, it has been proposed that VirS may be an SP receptor in C. perfringens (9). However, this important hypothesis had never been directly tested.

Given that CPB production is positively regulated by both the Agr-like QS system and the VirS/R TCRS (19, 25, 33), the current study used production of this toxin as a readout for SP signaling. Consequently, initial experiments in this study compared the timing of *cpb*, *agrD*, and *virS* expression in C. perfringens. Consistent with our previous reports that CPB is most highly produced during late log-phase growth by type B or C isolates in TGY medium (28, 29), the current study determined that, in TY cultures of either type B strain CN1796 or type C strain CN3685, *cpb* transcript levels increased from 1 h until they reached a peak at 5 h, a time corresponding to the late log phase-early stationary phase. Some transcription of *agrD* was observed even during early growth, but this expression also peaked at ∼5 h, consistent with previous studies showing that CPB production is controlled by the Agr-like QS system (25). In contrast, the *virS* gene was most strongly expressed early during growth, as would be expected if significant amounts of VirS membrane sensor already need to be available when the Agr-like QS maximally signals to increase CPB production.

We then conducted a series of studies to test directly whether VirS could be an SP receptor involved in regulating CPB production by C. perfringens type B or C strains. Since previous results (27) showed that an *agrB* mutant of type C strain CN3685 upregulates CPB production in response to both the 5R and 8R synthetic peptides but that an *agrB* mutant of type B strain CN1795 upregulates CPB production only in response to 5R, we reasoned that if VirS is a major SP receptor, there should be sequence differences between the VirS membrane sensor histidine kinase of these two strains. Sequencing did identify several differences between the deduced VirS amino acid sequences of these two strains, as shown in Table 1. When computer modeling was performed to predict the structural impact of those sequence variations, several structural differences were predicted, including differences in the predicted VirS ECL2. Results of experiments performed in this study then supported a role for the second ECL loop in SP binding. For example, preincubation of a synthetic peptide corresponding to ECL2 with 5R, the likely natural SP, inhibited signaling to increase CPB production in *agrB* mutants of CN3685 or CN1795.

This second ECL loop is predicted by computer modeling to include 14 amino acids (extending from amino acids 111 to 124) in the VirS made by CN1795 but to be 19 amino acids long (extending from amino acids 110 to 128) in the VirS made by CN3685. We speculate that, relative to the VirS made by CN1795, the larger size of its ECL2 allows the CN3685 VirS to functionally dock and accommodate the larger 8R (as well as the likely natural 5R) for signaling. Our results with ECL2 peptides (discussed in further detail below) implicated the Asp residue present at residue 117 in the CN1795 VirS as being important for SP binding. Detailed analysis of this loop should be performed in the future, since it represents a potential therapeutic target.

The VirS variant made by CN3685 is unusual. GenBank analysis of deduced VirS sequences in 53 other strains representing many C. perfringens types and origins indicated (data not shown) that only 2 of 53 strains resemble CN3685 in possessing a 6-amino-acid insertion at amino acid 152. This analysis also revealed that the KINSLVNVSELLGK ECL2 sequence of the CN3685 and CN1795 VirS was reasonably conserved among all 53 strains, with only 3 strains having 4 mutations and 26 strains having a single mutation. More common ECL2 sequence variations in these 26 strains were 16 strains producing an ECL2 with an S (versus a V) at position 6 and 10 strains producing an ECL2 with a D (versus an E) at position 10. Whether those ECL2 variations affect SP signaling should be investigated in the future.

Those findings also extend understanding of the VirS structure versus function relationship. Consistent with the modeling shown in Fig. 2, previous modeling work had also reported that the VirS sensor histidine kinase likely contains seven transmembrane (TM) domains and a C-terminal tail (13). That previous study (13) using random mutagenesis did not identify a putative SP binding site. However, it did determine that the predicted TM4 domain of the VirS N-terminal region is important for PFO production, which is regulated by both the VirS/R TCRS and the Agr-like QS system (9, 16, 23, 24, 34). Since modeling predicts that TM4 is adjacent to the second ECL, our new findings may suggest that the TM4 domain perturbations that altered PFO production in that previous study (13) affected the second ECL structure and thus altered SP binding and signaling. The previous random mutagenesis study (13) also identified regions of the C-terminal tail of VirS that are important for signaling, including the H255 residue that is an autophosphorylation site and G and N boxes that are involved in ATP binding and catalysis. The current results now add to that previous VirS structure versus function information by implicating the second ECL in SP binding and signaling.

Considerable progress was achieved toward the main goal of the study, i.e., testing the hypothesis that VirS is an SP receptor in C. perfringens. Several lines of evidence were obtained that now directly support VirS as an SP receptor. First, we exploited the previous observation (27) that both 5R and 8R can signal *agrB* null mutants of CN3685 while 5R (but not 8R) signals *agrB* null mutants of CN1795, which suggested that there may be differences between the VirS of CN3685 versus CN1795 if VirS is a major SP receptor. As discussed earlier, sequencing confirmed significant differences between the second ECL of VirS of CN3685 versus CN1795; therefore, we reasoned that, if VirS is an SP receptor, then swapping expression of these *virS* variants between *virR/S* and *agrB/D* double-null mutants of CN3685 versus those of CN1795 should change their sensitivity to 8R signaling. This hypothesis was supported when the CN1795 double mutant complemented to express the CN3685 VirS variant was shown to increase its CPB production in the presence of 8R, while the CN3685 double mutant complemented to express the CN1795 VirS variant lost the ability to increase CPB production in the presence of 8R. There was some CPB production by these complementing strains even in the absence of 8R; as noted previously, this effect is likely due to overexpression of VirR from the multicopy plasmid used for complementation (13).

A second approach to assess whether VirS is an SP receptor used a pulldown approach to evaluate directly if VirS can physically bind SP. Because attempts to prepare a VirS antibody were unsuccessful, this pulldown experiment instead used streptavidin beads pretreated with biotin-labeled 5R, which retains signaling activity. When those beads were reacted with extracts from CN3685 expressing a His_6_-tagged VirS, Western blotting with an His_6_ antibody detected specific pulldown of His_6_-tagged VirS using the biotin-labeled 5R beads. This result supported the ability of 5R to physically bind with VirS.

A third and final line of evidence supporting VirS as an SP receptor came from experiments using synthetic peptides corresponding to either the predicted ECL2 of VirS or to this ECL sequence with a single N to D substitution. The rationale behind this experiment was that if the second ECL of VirS is important for SP binding, as suggested by the VirS sequencing results described earlier, then preincubating this ECL2 peptide with 5R may cause ECL2:5R binding and thus inhibit subsequent Agr-like QS signaling by *agrB* mutants. This hypothesis was verified when preincubation of 5R with KIGK, the peptide corresponding to the wild-type ECL2 sequence of the CN1795 VirS, inhibited the ability of 5R to signal the *agrB* mutants of either CN3685 or CN1795, as evidenced by a reduction in 5R-induced CPB production. Furthermore, the presence of the KIGK peptide in wild-type CN3685 or CN1795 cultures also reduced CPB production. These effects were specific, since KIGK_D, which is the peptide corresponding to a single N to D substitution in KIGK, did not reduce the 5R-induced increase in CPB production by the same wild-type strains or their *agrB* mutants. In addition to supporting SP binding to VirS, these results (as mentioned earlier) also implicate the second ECL of VirS in this binding. Collectively, these ECL2 peptide results not only further support VirS:SP binding being a mechanistic basis for interactions between the Agr-like QS system and VirS/R TCRS during C. perfringens pathogenesis but could also be instructive for developing peptide therapeutics to inhibit QS signaling and reduce toxin production to control type C or type B diseases in which CPB is, respectively, proven or likely to be important for virulence.

The current findings coupling the Agr-like QS system and VirS/R TCRS also hold broader relevance for understanding C. perfringens pathogenesis beyond diseases caused by type B or C strains. Both the Agr-like QS system and VirS/R TCRS positively regulate production of CPA and PFO (9, 16, 23, 24), which are the toxins causing gas gangrene (35), and the importance of the Agr-like QS system for gas gangrene has been directly demonstrated (36). Similarly, both the Agr-like QS system and VirS/R control NetB toxin production, which is required for type G strains to cause avian necrotic enteritis, and the virulence of NetB-producing type G strains requires the Agr-like QS (17, 26). Therefore, coupling the Agr-like QS system and VirS/R TCRS provides C. perfringens with a single, versatile regulatory pathway for controlling production of several important toxins produced during vegetative growth. However, the Agr-like QS and VirS/R systems do not universally regulate all C. perfringens toxin production during vegetative growth. For example, *agrB* null mutants of type B strains CN1793 and CN1795 still produce wild-type levels of ETX (30). Regulatory control of ETX production is poorly understood and requires further study. Coupling of the Agr-like QS and VirS/R TCRS may also be important for regulating production of toxins produced during C. perfringens sporulation, since the Agr-like QS system is an important positive regulator of sporulation and C. perfringens enterotoxin (CPE) production by type F strains (37). However, it has not yet been assessed whether this regulation involves VirS/R.

While this study provides compelling support for VirS as an SP receptor, it remains possible that C. perfringens possesses one or more additional receptors for the SP of the Agr-like QS, particularly since this bacterium possesses ∼20 different TCRS (38). Supporting that possibility, silver staining of gels in the pulldown experiments revealed an ∼75-kDa band that was not reactive with His_6_ antibody and that had a larger molecular mass (∼75 kDa) than VirS (∼50 kDa) (data not shown). Whether that band reflects nonspecific binding or a second SP receptor will require further study.

In the S. aureus Agr system, SP signaling upregulates expression of the *agr* operon encoding itself and the AgrC receptor in a positive feedback loop (7, 8). Therefore, having obtained strong evidence supporting VirS as an SP receptor, we used qRT-PCR to examine whether the VirR/S TCRS and the Agr-like QS system upregulate expression of genes encoding each other. Results indicated that, in both CN3685 and CN1795 backgrounds, the presence of a functional *virS* gene results in significantly higher expression of the a*grD* gene and vice versa. This positive feedback effect was not attributable to growth differences between the mutants versus their wild-type parents. These observations suggest a model where small amounts of AgrD present early during growth may help to upregulate VirS production, which then results in the availability of more receptors to further amplify AgrD production and Agr-like QS signaling later during the growth cycle. This effect could contribute to toxin production and C. perfringens pathogenicity.

## MATERIALS AND METHODS

### Bacteria, media, and reagents.

C. perfringens wild-type, null mutant, and complementing strains used in this study are listed in Table 3. All isolates were stored in cooked meat medium (CMM) at −20°C. Fluid thioglycolate medium (FTG; Difco Laboratories), TY broth (3% tryptic soy broth [Becton, Dickinson], 1% yeast extract [Becton, Dickinson], and 0.1% sodium thioglycolate [Sigma-Aldrich]), and TGY broth (TY broth supplemented with 2% glucose [Sigma-Aldrich]) were used for broth cultures. After inoculation, brain heart infusion (BHI) agar (Research Products International) plates containing 15 μg · ml^−1^ chloramphenicol (Sigma-Aldrich) were incubated at 37°C under anaerobic growth conditions in GasPak jars to screen the knockout mutants constructed in this study. A CN3685::*agrB* null mutant named BMJV10, a CN1795::*agrB* null mutant, and complementing strains of both mutants had been previously constructed and characterized (25, 30). All chemical reagents used in this study were purchased from Fisher Scientific, Sigma-Aldrich, or Bio-Rad Laboratories.

**TABLE 3:** C. perfringens strains used in this study

Type or isolate	Description	Origin and/or reference
Type B		
CN1795	Veterinary lab, toxigenic, isolated in 1947	28
CN1795::*agrB*	CN1795 *agrB* null mutant	28
CN1795::*agrB*comp	CN1795 *agrB* null mutant complemented with *agr* operon	28
CN1795::*virS*	CN1795 *virS* null mutant	This study
CN1795::*virS*c1795*virR*/*S*	CN1795 *virS* null mutant complemented with CN1795 *virR/S* operon	This study
CN1795::*virS*c3685*virR*/*S*	CN1795 *virS* null mutant complemented with CN3685 *virR/S* operon	This study
CN1795DKO	CN1795 *virS agrB* double-null mutant	This study
CN1795DKOc3685*virR*/*S*	CN1795 *virS agrB* double-null mutant complemented with CN3685 *virR/S* operon	This study
Type C		
CN3685	Sheep with the disease named struck	25
CN3685::*agrB* (BMJV10)	CN3685 *agrB* null mutant	25
CN3685::*agrB*comp	CN3685 *agrB* null mutant complemented with *agr* operon	25
CN3685::*virS*	CN3685 *virS* null mutant	This study
CN3685::*virS*c1795*virR*/*S*	CN3685 *virS* null mutant complemented with CN1795 *virR/S* operon	This study
CN3685::*virS*c3685v*irR*/S	CN3685 *virS* null mutant complemented with CN3685 *virR/S* operon	This study
CN3685DKO	CN3685 *virS agrB* double-null mutant	This study
CN3685DKOc1795v*irR*/*S*	CN3685 *virS agrB* double-null mutant complemented with CN1795 *virR/S* operon	This study

### Synthetic peptides.

The synthesis of all peptides used in this study was carried out by the Peptide and Peptoid Synthesis Core Facility Division of the Health Sciences Core Research Facilities (HSCRF) at the University of Pittsburgh. SP-based synthetic peptides used included (i) 5R, a 5-mer cyclic ring likely corresponding to the wild-type C. perfringens SP (27); (ii) 8R, an 8-mer consisting of 5R plus a 3-amino-acid tail; and (iii) Bio-5R, a biotin-labeled version of 5R. These peptides were prepared as described in previous studies (27). Synthesis of the linear VirS-based peptides KIGK and KIGK_D (Table 2) were performed on a 50 μM scale using standard 9-fluorenylemethyloxycarbonyl (FMOC) chemistry cycles on a Liberty Blue CEM microwave synthesizer using oxyma/DIC [ethyl-(2Z)-2-cyano-2-hydroxyiminiacetate/*N*,*N*-diisopropylcarbodiimide) activation in dimethylforamide (DMF). FMOC-protected amino acids (EMD Millipore) were used for the stepwise assembly of these linear sequences on preloaded FMOC-Lys(Boc) Wang resin (0.56 mM; Peptides International). A cleavage cocktail (90% trifluoroacetic acid [TFA], 5% thioanisole, 3% 3,6-dioxa-1,8-octanedithiol [DODT], and 2% anisole) was used for 4 h at room temperature, while shaking, to cleave KIGK and KIGK_D from the Wang resin as well as to scavenge side chain-protecting groups. These peptides were first precipitated in cold diethyl ether and then washed and centrifuged 3 times with additional diethyl ether, resulting in a pellet of crude peptide. Crude KIGK and KIGK_D were allowed to air dry and were then dissolved in 0.1% TFA, which was then frozen and lyophilized overnight to remove remaining organics. Each crude peptide was dissolved in 0.1% TFA and then directly loaded onto a Waters 2555 Quaternary Gradient Module with a Waters 2489 UV-visible (UV-Vis) detector and purified on a Phenomenex Gemini (250 mm × 21.20 mm) 10-μm C_18_ column using standard acetonitrile (ACN)/0/1% TFA gradient conditions. Final analytical determinations of peptide purity for KIGK and KIGK_D were performed on a Waters e2695 separations module with a Waters 2489 UV-Vis detector using a Phenomenex Gemini-NX (250 mm × 4.6 mm) 5 μm C_18_ column using standard acetonitrile (ACN)/0.1% TFA gradient conditions. Mass spectrometry analysis using an Applied Biosystems Voyager DE-STR matrix-assisted laser desorption ionization–time of flight (MALDI-TOF) was used to confirm the expected mass of each peptide, as follows: KIGK expected, 1,513.80; observed, 1,513.04; KIGK_D expected, 1,514.78; observed 1,514.06.

Each synthetic peptide was resuspended in dimethyl sulfoxide (DMSO; Fisher Scientific) at 50 mM before use. Aliquots (2.0, 10, or 20 μl) of this suspension of synthetic peptide in DMSO were then added to 1 ml of culture medium, resulting in a final peptide concentration of 0.1, 0.5, or 1 mM. After a 5-h or overnight (16-h) culture, CPB or His-tagged Western blots were performed using culture supernatants or pelleted cells (see “Western blot analyses of CPB production”).

### Sequencing of the *virR/S* operon in CN1795 and CN3685.

DNA was isolated from CN1795 or CN3685 using the MasterPure Gram-positive DNA purification kit (Epicentre). The primers used in a PCR to amplify this operon are listed in Table 4. For this PCR, 0.25 μl of each primer (at a 25 μM final concentration), 1 μl of purified DNA template, and 25 μl NEBNext high-fidelity 2× PCR mastermix (New England Biolabs) were mixed together, and double-distilled water (ddH_2_O) was then added to reach a total reaction volume of 50 μl. The reaction mixtures were placed in a thermal cycler (Techne) and then subjected to the following amplification conditions: 1 cycle of 98°C for 30 sec; 35 cycles of 98°C for 10 s, 50 to 72°C (depending on the primers used) for 20 s, and 72°C for 2 min, followed by a single extension of 72°C for 2 min. The resultant PCR products were cleaned up using a QIAquick PCR purification kit (Qiagen) and sent for DNA sequencing to the University of Pittsburgh Core Sequencing Facility.

**TABLE 4:** Primers used in this study

purposePrimer purpose	Primer name	Primer sequence	PCR product size (bp)
pJIR750virSi construction	virS-293|294a-IBS	AAAAAAGCTTATAATTATCCTTATCTACCGTAAGCGTGCGCCCAGATAGGGTG	350
	virS-293|294a-EBS1d	CAGATTGTACAAATGTGGTGATAACAGATAAGTCGTAAGCATTAACTTACCTTTCTTTGT	
	virS-293|294a-EBS2	TGAACGCAAGTTTCTAATTTCGGTTGTAGATCGATAGAGGAAAGTGTCT	
	EBS universal	CGAAATTAGAAACTTGC GTTCAGTAAAC	
Screen for intron insertion in *virS* (*virS* RT-PCR)	virSKOF	TAAGTCAATTTAGCCCTAAGAAAA	376
	virSKOR	CGAAAC TTTAAACATCTAACAACCA	
Screen for intron insertion in *agrB* (*agrB* RT-PCR)	NagrBKOF	TGGAACTTATGCTCTAATACAAACA	536
	agrBKOR	AATCTATAGTTTTTAACAATATATTT	
*cpb* qRT-PCR	cpbF	GCGAATATGCTGAATCATCTA	196
	cpbR	GCAGGAACATTAGTATATCTTC	
*virS* qRT-PCR	qvirSF	CATAGCCTGTATTGAAGGAAATAAC	229
	qvirSR	TGTGCAGATATCAAAGTACTCA	
*virR* qRT-PCR	qvirRF	CCTTTGAGACAGGAGAGGATCTA	240
	qvirRR	CCTGCTCTTGTAGCTCCTTAAAT	
*agrD* qRT-PCR	qagrDF	GCTGCATTAACAACAGTAGTTGC	76
	qagrDR	GTTCCTCTGGTTGGTGTGTAAA	
*virR/S* operon sequencing	VirR/SseqF	GTCTCAAAGATCTAGTAAAATGGGA	838
	VirR/SseqR	GCCCTTATTATTAAGCTCCTTTTCT	
	VirR/Sseq1	CAGGTTACAGCTTGTGTAGAAAATA	912
	VirR/Sseq1R	CCTTAAAGGCATATCCAAATATAAC	
	VirR/Sseq2	CAATATAAAATGTATTATGATCTC	1,050
	VirR/Sseq2R	GGAATGAGCATTTTTAATATGAATT	
	VirR/Sseq3	GACAAGCTAAACTTAGGATT	813
	VirR/Sseq3R	TTCCATTTACCTGAATTAACTCACT	
*virR/S* complementation	VirR/ScompF	CCGGGGATCCGAAAGTGGATATGCACTAGGAAC	2,494
	VirR/ScompR	ATGCCTGCAGTGCAAAGCTTAAAACTGTAACTGTA	
*virR/S*-His tag complementation	VirR/ShisR	ATGCCTGCAGTTAATGATGATGATGATGATGGGCTTCTTTTTCTTGATTTATAGG	2,249

### Computer modeling of the VirS protein structure.

The *virS* sequencing results were used to model the predicted structure of VirS made by CN1795 or CN3685 using the TMHMM server v.2.0 (prediction of transmembrane helices in proteins; http://www.cbs.dtu.dk/services/TMHMM/) transmembrane prediction algorithms.

### Plasmids and primers.

A plasmid named pJIR750virSi was constructed to inactivate, using Clostridium-modified group II TargeTron technology (31), the *virS* gene and create single *virS* null mutants or, using existing *agrB* mutants, double mutants unable to express both *virS* and the operon encoding AgrB/D. The primers used for intron targeting to the *virS* gene were virS-293|294a-IBS, virS-293|294a-EBS1d, virS-293|294a-EBS2, and EBS universal primers. All primer sequences are listed in Table 4. The 350-bp intron PCR product was then inserted into pJIR750ai (31) between the HindIII and BsrGI enzyme (New England Biolabs) cut sites to construct the pJIR750virSi vector. The *virS* null mutant screening primers used were virSKOF and virSKOR. The same pair of primers were also employed to analyze *virS* gene expression. The primers used for PCR amplification of the *agrB* gene or for *agrB* gene RT-PCR were NagrBKOF and agrBKOR. The qRT-PCR primers used in this study were designed and synthesized by Integrated DNA Technologies (IDT). These primers were specific for 16S RNA (as a control housekeeping gene) (39), the *virS* gene (qvirSF and qvirSR), or the *agrD* gene (qagrDF and qagrDR).

A *virR/S* operon complementation vector named pJIR750virR/Scomp was constructed as follows. A region of CN1795 or CN3685 DNA spanning from ∼200 bp upstream of the *virR/S* operon to ∼200 bp downstream of the *virR/S* operon was PCR amplified using primers VirR/ScompF and VirR/ScompR (Table 4). The resultant PCR product (∼3,000 bp) was then ligated into pJIR750 between the BamHI and PstI restriction sites, followed by electroporation of the *virR/S* operon-carrying plasmid pJIR750virR/Scomp into the CN1795::*virS*, CN3685::*virS*, CN1795DKO or CN3685DKO mutants (see Results). Transformants were then selected on BHI agar plates containing 15 μg · ml^−1^ of chloramphenicol with anaerobic incubation in GasPak jars. For expression of His_6_-tagged VirS, the *virR/S* operon was PCR amplified using primers VirR/ScompF and VirR/ShisR (Table 4), which added a His_6_ sequence to the C-terminal end of the VirS membrane protein. The resultant PCR product was then ligated into pJIR750 between the BamHI and PstI restriction sites to create the plasmid pJIR750virS/hiscomp. This plasmid was then electroporated into CN3685::*virS* to create a complementing strain (CN3685::*virS*c3685*virS*his) that expresses VirS protein labeled with a His_6_ tag.

### Construction of CN1795 and CN3685 *virS* single-null mutants and complemented strains or double-null mutants of those strains with inactivated *virS* and *agrB* genes.

The *virS* gene was disrupted in CN1795 or CN3685 to generate single-null mutant strains that do not produce VirS. The *virS* gene was also inactivated in existing CN1795::*agrB* or CN3685::*agrB* strains (25, 30) to create double-null mutants with inactivated *virS* and *agrB* genes. Disruption of the *virS* gene in these mutants was achieved by specifically inserting, in the antisense orientation, a group II intron (∼900 bp) into the *virS* gene, generating *virS* single-null mutants or *virS agrB* double-null mutants of CN1795 or CN3685. For this purpose, the intron donor plasmid pJIR750virSi, which carries a *virS*-targeted intron, was electroporated into CN1795, CN3685, CN1795::*agrB*, or CN3685::*agrB*. Transformants were selected by plating onto BHI agar plates containing 15 μg · ml^−1^ of chloramphenicol, followed by overnight anaerobic growth in a GasPak jar. Colony PCR was carried out for screening using internal *virS* primers virSKOF and virSKOR (Table 4), which amplify a PCR product of ∼370 bp using DNA from a wild-type strain but amplify an ∼1,300-bp product using DNA from *virS* null mutants due to the insertion of an ∼900 bp intron. Each *virS* gene null mutant was subcultured daily in FTG medium over 10 days to cure the intron-carrying plasmid, creating a CN1795 *virS* null mutant (CN1795::*virS*), a CN3685 *virS* null mutant (CN3685::*virS*), a CN1795 *virS agrB* double-null mutant (CN1795DKO), and a CN3685 *virS agrB* double-null mutant (CN3685DKO). Each mutant was then further characterized by PCR, RT-PCR, and Southern blotting analyses, as described below.

VirS complementing strains of the single and double mutants were prepared by transformation with pJIR750*virR*/*S1795comp* or pJIR750*virR*/*S*3685comp. Transformants were selected on chloramphenicol, as described earlier.

### Measurement of C. perfringens growth.

For analysis of C. perfringens vegetative growth, a 0.2-ml aliquot of an overnight FTG culture of a wild-type or null mutant strain was inoculated into 10 ml of TY medium. The cultures were incubated at 37°C; thereafter, at 0-, 1-, 3-, 5-, 8-, and 24-h culture times, 1 ml of each culture was removed for measurement of optical density at 600 nm (OD_600_) using a Bio-Rad Smart spectrometer.

For the two wild-type strains, another 1-ml aliquot of culture was removed and centrifuged at 15,000 rpm for 3 min. Equal volumes of each resultant culture supernatant were then mixed with 5× SDS loading buffer and boiled for 5 min. An aliquot (30 μl) of each boiled sample was electrophoresed on a 10% SDS-PAGE gel and then subjected to a CPB Western blot analysis (see “Western blot analyses of CPB production”). Using the same cultures, total RNA was isolated from the pellets at each time point, and qRT-PCR were performed as described in “C. perfringens RNA isolation, RT-PCR, and qRT-PCR analyses.”

### C. perfringens DNA isolation, PCR, and intron Southern blot analyses.

DNA was extracted from all C. perfringens strains using the MasterPure Gram-positive DNA purification kit. PCR for the *virS* or *agrB* genes was performed using the primers described in the previous section. For the wild-type strains, the sizes of PCR products are listed in Table 4. For the null mutant strains, PCR using the same pair of the primers should amplify a product from the intron-disrupted gene that is ∼900 bp larger than the gene from the wild-type strains. All PCR conditions were described previously (25, 30).

For Southern blot analysis to detect an intron insertion, aliquots (3 μg each) of wild-type, single-null, or double-null mutant strain DNA were digested overnight with EcoRI at 37°C according to the manufacturer’s instructions (New England Biolabs). The digested DNA samples were then electrophoresed on a 1% agarose gel before transfer onto a positively charged nylon membrane (Roche) for hybridization with an intron-specific probe (20). The intron-specific probe was prepared using the PCR digoxigenin (DIG) probe synthesis kit (Roche) and intron primers (IBS and EBS2). After hybridization, Southern blots were developed using reagents from the DIG DNA labeling and detection kit (Roche) according to the manufacturer’s instructions.

### C. perfringens RNA isolation, RT-PCR, and qRT-PCR analyses.

All tested isolates were grown in TY broth for 2 h (for analyzing *virS* expression) or 5 h (for analyzing expression of *agrD* and *agrB*) at 37°C. Cultures were then pelleted at the indicated times, and RNA was extracted using saturated phenol and purified by TRIzol and chloroform (Life Technologies and Sigma), as previously described (39). Before RT-PCR or qRT-PCR analysis was performed, the isolated RNA was subjected to regular PCR without reverse transcriptase to confirm that samples were free of DNA. If the sample had any DNA contamination, DNase (Thermo Fisher) was used to remove the residual DNA. RNA was then quantified by determining the absorbance at 260 nm. The purified RNA was used to prepare cDNA or stored in a −80°C freezer for further experiments.

A 1-μl aliquot of purified RNA (100 ng) was used in a one-step RT-PCR containing 10 μl of 2× *Taq* mastermix (New England Biolabs), avian myeloblastosis virus (AMV) reverse transcriptase (4 U; Promega), ddH_2_O, and primers specific for the *virS* or *agrB* genes (Table 4), 16S RNA RT-PCR was performed to serve as a loading control (39). Reaction mixtures were incubated for 45 min at 45°C to allow cDNA synthesis, then regular PCR cycling was performed as follows: (i) 95°C for 2 min; (ii) 30 cycles of 95°C for 15s, 50°C for 30s, and 68°C for 30s; and (iii) a final extension of 68°C for 5 min.

For qRT-PCR, an aliquot (500 ng) of purified RNA in a total 10-μl reaction mixture was first synthesized to cDNA using a Maxima first-strand cDNA synthesis kit (Thermo Scientific), according to the manufacturer’s instructions. The cDNA synthesis programming was as follows: 25°C for 10 min, 50°C for 30 min, and 85°C for 5 min. All qRT-PCR primers were designed using the Integrated DNA Technologies (IDT) website and are listed in Table 4. Each cDNA was diluted 10 times to 5 ng/μl. Power SYBR green PCR master mix (Thermo Fisher Scientific) and a StepOnePlus qRT-PCR instrument (Applied Biosystems) were used to perform qRT-PCR as described in a previous paper (39). After qRT-PCR, the relative quantitation of mRNA expression was normalized to the level of constitutive expression of the housekeeping 16S RNA and calculated by the comparative threshold cycle (2^−ΔΔ^*^CT^*) method (39).

### Use of SP-based peptides to induce CPB production.

Wild-type or *agrB* mutants of CN1795 and CN3685 were each grown in 10 ml of FTG overnight at 37°C. An aliquot (0.2 ml) of each culture was inoculated into 10 ml of fresh TY broth, and those cultures were incubated overnight (about 16 h) at 37°C. A 15-μl aliquot of each TY overnight culture was then inoculated into 1 ml of TY medium in a 1.5-ml microcentrifuge tube with a screw cap (Fisher Scientific) that contained a final concentration of 100 μM and SP-based peptide (5R, 8R, or B-5R) suspended in DMSO. As a control, an equal volume of DMSO alone (no peptide) was added to some cultures. These cultures were incubated for 5 h or overnight at 37°C as specified in the experiments.

### Use of VirS ECL2-based peptides to inhibit CPB signaling.

CN1795 and CN3685 *agrB* null mutant strains were each grown in 10 ml of FTG medium overnight at 37°C. An aliquot (0.2 ml) of each culture was inoculated into 10 ml of fresh TY broth, and those cultures were incubated overnight (about 16 h) at 37°C. Before the addition of a 15-μl aliquot of each TY overnight culture, 5R (100 μM final concentration), together with a 500 μM final concentration of the 14-amino-acid peptide (KIGK) corresponding to the VirS ECL2 sequence, the variant peptide (KIGK_D), or a control peptide, was dissolved in DMSO and the mixed-in 1 ml TY medium and incubated at 37°C for 30 min. A 15-μl aliquot of the overnight *agrB* null mutant culture was inoculated into this medium and incubated for 5 h. At that time, the culture supernatants were collected and used for CPB Western blot analysis.

In a second experiment, wild-type CN1795 and CN3685 cultures were each grown in 10 ml of FTG overnight at 37°C. An aliquot (0.2 ml) of each culture was inoculated into 10 ml of fresh TY broth, and those cultures were incubated overnight (about 16 h) at 37°C. A 15-μl aliquot of each TY overnight culture was then inoculated into 1 ml of TY medium in a 1.5-ml microcentrifuge tube with a screw cap that contained a final concentration of 500 μM or 1 mM KIGK or KIGK_D dissolved in DMSO. These cultures were then incubated for 5 h. At that time, the culture supernatants were collected and used for CPB Western blot analysis.

### Western blot analyses of CPB production.

Aliquots of each culture were adjusted to equal OD_600_ values and then centrifuged. The supernatants were mixed with 5× SDS-PAGE loading buffer and boiled for 5 min. Aliquots (20 μl) of each sample were electrophoresed on a 10% SDS-PAGE gel, and the separated proteins were then transferred onto a nitrocellulose membrane. The membrane was blocked with TTBS, i.e., Tris-buffered saline-Tween 20 (0.05% vol/vol), and nonfat dry milk (5% wt/vol) for 1 h at room temperature, followed by probing with a rabbit poly-anti-CPB antibody (1:1,000 dilution) overnight at 4°C. Finally, bound antibody was detected with a horseradish peroxidase-conjugated secondary anti-rabbit antibody (Sigma-Aldrich), followed by addition of SuperSignal West Pico chemiluminescent substrate (Fisher Scientific).

### His_6_-tagged VirS pulldown by Bio-5R and detection by Western blotting.

CN3685::*virS* was transformed with a complementation vector named pJIR750virR/Shiscomp, which encodes a His_6_-tagged VirS (see “Plasmids and primers”), to create CN3685::*virS*c3685*virR*/*S*his. As an antibody specificity control, CN3685::*virS* was also transformed to create a strain named CN3685::*virS*c3685*virR*/*S* that expresses untagged VirS. A 0.2-ml aliquot of a CMM stock of each culture was transferred to 10 ml of FTG medium and then cultured overnight at 37°C. A 0.2-ml aliquot of each FTG culture was transferred to 10 ml of TY medium for another overnight culture at 37°C. A 2-ml aliquot of this overnight TY culture was transferred to 100 ml of fresh TY medium and cultured for 4 h at 37°C. Those cultures were then centrifuged and the pellets frozen at −80°C for at least 2 h. The frozen pellets were resuspended in 1 ml of B-PER buffer (Fisher Scientific) with proteinase inhibitor (Research Products International) at room temperature for 30 min with gentle shaking. At the same time, a 100-μl aliquot of streptavidin MagBeads (GenScript) was washed twice with B-PER buffer. The washed beads were then incubated with either biotinylated 5R (Bio-5R) or control 5R in 500 μl of B-PER buffer (200 μM) for 1 h at room temperature with slow end-over-end mixing. These pretreated beads were then washed twice with B-PER buffer. The bacterial supernatants, prepared as described above, were applied to the washed MagBeads, and the mixture was incubated at 4°C overnight with slow end-over-end mixing. The MagBeads were washed three times and then 80 μl of 2× SDS loading dye was added, followed by boiling for 5 min. A 40-μl aliquot of each sample was loaded on a 10% SDS gel, followed by a His_6_ tag detection Western blot. For this purpose, a His6 tag antibody (Aviva Systems Biology OAEA00010) was purchased from Fisher Scientific. The antibody was used at a 1:1,000 dilution in TTBS with 5% milk. Finally, bound antibody was detected with a horseradish peroxidase-conjugated secondary anti-mouse antibody (Sigma-Aldrich), followed by addition of Clarity and Clarity Max ECL Western blotting substrates (Bio-Rad).

### Statistical analyses.

All statistical analyses were performed using GraphPad Prism 8. For comparison of more than two samples, one-way analysis of variance (ANOVA) was applied with *post hoc* analysis by Dunnett’s multiple-comparison test. For comparison of two samples, Student’s *t* test was applied. Differences were considered significant when the *P* value was less than 0.05.

### Data availability.

The sequence results were submitted to NCBI GenBank (accession numbers MT597430 and MT597431).
